# Electrical Conductivity Based Ammonia Sensing Properties of Polypyrrole/MoS_2_ Nanocomposite

**DOI:** 10.3390/polym12123047

**Published:** 2020-12-18

**Authors:** Sharique Ahmad, Imran Khan, Ahmad Husain, Anish Khan, Abdullah M. Asiri

**Affiliations:** 1 Applied Science and Humanities Section, University Polytechnic, Faculty of Engineering and Technology, Aligarh Muslim University, Aligarh 202002, India; shariqueahmad14@gmail.com (S.A.); imrannano@gmail.com (I.K.); 2 Department of Applied Chemistry, Faculty of Engineering and Technology, Aligarh Muslim University, Aligarh 202002, India; ahmadhusain2065@gmail.com; 3 Center of Excellence for Advanced Materials Research, King Abdulaziz University, Jeddah 21589, Saudi Arabia; 4 Chemistry Department, Faculty of Science, King Abdulaziz University, Jeddah 21589, Saudi Arabia

**Keywords:** nanocomposite, polypyrrole/MoS_2_, ammonia sensing, electrical conductivity

## Abstract

Polypyrrole (PPy) and Polypyrrole/MoS_2_ (PPy/MoS_2_) nanocomposites were successfully prepared, characterized and studied for ammonia sensing properties. The as-prepared PPy and PPy/MoS_2_ nanocomposites were confirmed by FTIR (Fourier transform infrared spectroscopy), XRD (X-ray diffraction), SEM (scanning electron microscopy) and TEM (transmission electron microscopy) techniques. The ammonia sensing properties of PPy and PPy/MoS_2_ nanocomposites were studied in terms of change in DC electrical conductivity on exposure to ammonia vapors followed by ambient air at room temperature. It was observed that the incorporation of MoS_2_ in PPy showed high sensitivity, significant stability and excellent reversibility. The enhanced sensing properties of PPy/MoS_2_ nanocomposites could be attributed to comparatively high surface area, appropriate sensing channels and efficiently available active sites. The sensing mechanism is explained on the basis of simple acid-base chemistry of polypyrrole.

## 1. Introduction

A new era began in the field of polymers after the discovery of conductivity in polyacetylene by Shirakawa et al. [1]. Over the years, the field of conducting polymers has seen a great extent of research been carried out since it first came to light that conjugated polymers could be made to conduct by the process of doping. They have aroused great interest since then and have been employed to fabricate sensing devices as their electrical and electrochemical properties can easily and precisely be altered according to necessities [2,3,4,5,6,7,8,9,10]. In recent times, conducting polymers such as polythiophene (PTh), polypyrrole (PPy) and polyaniline (Pani) have been extensively used for the fabrication of gas and vapor sensors [2,3,4,8,9,10,11,12,13,14,15]. Among the several conducting polymers available nowadays, polypyrrole (PPy) and its composite materials have attracted attention as it is easy to synthesize, possesses high conductivity and is environmentally stable [6,9].

It is well-recognized that amalgamating polypyrrole with inorganic nanomaterials results in the formation of nanocomposites which are environmentally and chemically more stable than pristine PPy [6,9,12]. DC electrical conductivity and gas/vapor sensing properties of PPy can be significantly enhanced by the formation of its nanocomposites [12]. Usually, these nanocomposites have been synthesized by the oxidative chemical polymerization method and have been used for detecting a number of gases and volatile organic compounds (VOCs). The sensors based on conductive polymers (CPs) such as polypyrrole (PPy) offer great advantages, such as they are highly sensitive, have shorter response time and can operate at room temperature. These sensors display excellent sensing ability due to the synergistic effect of both the components, i.e., PPy and nano-fillers [6,9,12,16,17,18].

Herein, molybdenum disulfide (MoS_2_) is employed as a nano-filler in order to synthesize PPy/MoS_2_ nanocomposites for enhancing electrical and ammonia sensing properties of pristine PPy. Since its discovery by Tenne et al., MoS_2_ nanoparticles have been established as an outstanding material in electronics, optoelectronics and sensor chemistry due to its low band gap (1.8 eV) and thermal and chemical stability [19,20,21,22,23,24,25,26]. Therefore, MoS_2_ nanoparticles are utilized as a nano−filler for the preparation of conducting polymers’ nanocomposites. The incorporation of MoS_2_ nanoparticles into polymer matrix enhanced the electrical, optical, thermal and mechanical properties of polymers [5,27,28,29].

Among the various toxic gases/vapors, ammonia is the most commonly produced from vehicular emissions and various industries. It is very harmful if inhaled in large amounts, usually greater than 300 ppm, and may damage human body cells and various respiratory diseases. It may also cause various problems to human health such as sore throat, headache, chest pain, fatigue, vomiting, etc. Therefore, detection of ammonia at ppm levels is compulsory for the assessment of human health [2,4,16,17]. Conducting polymer-based materials have been utilized for the fast and selective detection of ammonia in the form of pellet-shaped sensors. Husain et al. fabricated a pellet-shaped ammonia sensor utilizing polythiophene/ multi walled carbon nanotubes and polythiophene/ single walled carbon nanotubes (PTh/MWCNT and PTh/SWCNT) nanocomposites which can detect it selectively at an extremely low concentration of 0.1 and 0.5 ppm, respectively [16,17]. Ahmad et al. reported a novel pellet-shaped ammonia sensor based on Pani/nickel oxide/graphene nanocomposites with lower detection limit of 170 ppm at room temperature [30].

Niaz et al. [31] successfully prepared a MoS_2_/PPy nanocomposite by the in-situ oxidative polymerization method. They evaluated its electrochemical properties for possible application in supercapacitors as electrode materials. The results showed that the MoS_2_/PPy nanocomposite electrode exhibited very high specific capacitance (654 Fg-1) along with 95% retention of performance, even after 500 cycles. Lian et al. [32] also utilized a PPy/MoS_2_ nanocomposite for the fabrication of electrode materials in supercapacitors. The results revealed that the electrode possesses an exceptionally high specific capacitance (895.6 Fg-1) as well as outstanding cycling stability of about 98% after 10,000 cycles. In another study, Lei et al. [33] synthesized the MoS_2_/PPy nanocomposite by the hydrothermal method. They reported that the MoS_2_/PPy nanocomposite showed greater peroxidase-like catalytic action towards oxidation of 3,3,5,5-tetramethylbenzidine (TMB) in the presence of hydrogen peroxide in acetate buffer solution. The catalytic performance of the MoS_2_/PPy nanocomposite was found to be superior to the pure PPy and MoS_2_.

Papadopoulou et al. synthesized a polyimide-based real-time ammonia sensor by measuring the changes in the electric current intensity with concentration as low as 3.5 mM [34].

To the best of our knowledge, this is the first report on ammonia sensing studies of the PPy/MoS_2_ nanocomposite. Thus, we firmly believe that this study will contribute significantly towards the fabrication of a highly sensitive, completely reversible and very selective gas/vapor sensor working at room temperature.

In this study, we have prepared pristine PPy and nanocomposites of PPy having 20 wt.% of MoS_2_ with respect to the weight of pyrrole (monomers). The successful formation of PPy and PPy/MoS_2_ nanocomposites have been confirmed by FTIR, XRD, SEM and TEM techniques. Electrical and ammonia sensing properties of the materials were studied at room temperature.

## 2. Materials, Methods and Instrumentation

### 2.1. Materials

Pyrrole 99% (Sigma-Aldrich, St. Louis, MI, USA), molybdenum disulfide (MoS_2_) (CDH, India), anhydrous ferric chloride (Fischer scientific India) and methanol (Fischer scientific India) were used. We used double-distilled water in the experiments.

### 2.2. Instrumentation

FTIR (Fourier transform infrared spectroscopy) was performed on different samples between a wavenumber range of 4000–400 cm^−1^ by the spectrophotometer Nicolet (Model iS50, Thermo Scientific, Berkeley, MI, USA) with a built-in ATR (Attenuated Total Reflectance) accessory. The JEOL model (JSM-7600F, JEOL, Tokyo, Japan).JSM-7600F was used for scanning electron microscopy (SEM) photographs’ collection, while the transmission electron microscopy (TEM) images were collected through the instrument JEOL model (JEM-6510LV, JEOL, Japan). XRD patterns of the solids were recorded at room temperature (RT) by a Thermo-Scientific powder diffractometer instrument (model ARL X’TRA, Thermo Scientific, Berkeley, MI, USA). The angle of the diffractometer was kept at a 2θ range from 10 to 80°, while the scanning rate was 0.002°s^−1^ with a Copper Kα radiation (λ = 1.5418 Å).

### 2.3. Preparation of PPy and PPy/MoS_2_ Nanocomposite

Polypyrrole (PPy) and PPy/MoS_2_ nanocomposites were prepared via the in-situ oxidative polymerization routine in the aqueous medium using anhydrous ferric chloride (FeCl_3_) as the oxidant [9,12]. Firstly, a requisite amount of MoS_2_ powder was added into a 500 mL beaker having 200 mL of double-distilled water, then ultra-sonicated for 4 h for the exfoliation of MoS_2_ sheets. After that, 4.16 ml (0.06 mol) of pyrrole monomers were transferred into the beaker containing MoS_2_ and further sonicated for 2 h for the adsorption of monomers on the surface of MoS_2_. Then, 9.72 g (0.06 mol) of anhydrous ferric chloride was added into a beaker containing 100 mL of double-distilled water and stirred for a few minutes. Afterward, the prepared aqueous solution of ferric chloride was transferred dropwise into the beaker containing pyrrole monomers and MoS_2_, along with continuous stirring for 10 h in order to achieve thorough polymerization of pyrrole. After polymerization, the nanocomposite was obtained as a black slurry which was filtered along with washing carefully with double-distilled water as well as methanol. Finally, washed and filtered material was dried in an oven at 70 °C for 12 h and converted into powder for characterization. The pristine PPy nanoparticles have also been prepared by the same method for reference and comparison.

### 2.4. Characterization 

The Perkin-Elmer-1725 instrument on KBr-pellets was used for recording FTIR spectra of PPy, MoS_2_ and PPy/MoS_2_. The morphology was determined by SEM and TEM images. The scanning electron microscopic studies were done to study the surface morphology by scanning a focused electron beam over the surface of the materials by JEOL, JSM, 6510-LV (JEOL, Japan). The transmission electron microscopic studies were done to study the detailed internal morphology of the material by JEM 2100, (JEOL, Japan). The crystalline and amorphous nature of the material was analyzed by X-rays diffraction patterns. XRD data of materials were recorded by a Bruker D8 diffractometer with Cu Kα radiation at 1.540 Å in the range of 5° ≤ 2θ ≤ 80° at 50 kV.

DC electrical conductivity as well as ammonia sensing experiments were carried out with four in-line probe instruments attached with a PID (proportional integral derivative)-controlled oven manufactured by Scientific Equipment, Roorkee, India. The equation used for the calculation of DC electrical conductivity is given below:

σ = [ln2 (2S/W)]/[2πS (V/I)](1)

where I, V, W, S and σ represent the current (A), voltage (V), the thickness of the pellet (cm), probe spacing (cm) and conductivity (S·cm^−1^), respectively [11,12,13]. The pellets used in conductivity and sensing experiments were made by a hydraulic pressure machine at 70 kN pressure applied for 60 s. 250 mg of each sample was used for the preparation of pellets. 

## 3. Results and Discussions

### 3.1. Fourier Transform Infrared Spectroscopic (FTIR) Studies 

**Figure 1:**
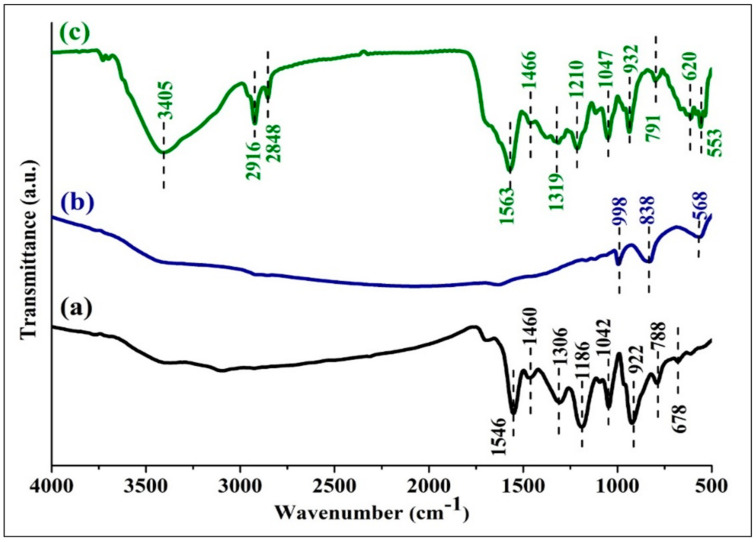
The Fourier transform infrared (FTIR) spectra of: (**a**) PPy, (**b**) MoS_2_ and (**c**) PPy/MoS_2_.

The FTIR spectra of PPy, MoS_2_ and PPy/MoS_2_ nanocomposites are depicted in Figure 1. The typical peaks of PPy observed at 1546 and 1460 cm^−1^ are due to C=C stretching vibrations, as shown in Figure 1a. The peaks at 1306, 1186 and 1042 cm^−1^ are related to the C=N bending, C−N stretching and =C−H bending vibrations of PPy, respectively. The two peaks at 788 and 678 cm^−1^ are attributed to C−H out of plane deformational vibration-mode of the PPy ring. The peak present at 922 cm^−1^ corresponds to the C=N^+^−C stretching vibration. This peak at 922 cm^−1^ confirms the doping of PPy by FeCl_3_ and creation of charge carriers, i.e., polarons [6,7,9,12,31]. 

In the spectrum of MoS_2_ (Figure 1b), the peaks witnessed at 998, 839 and 568 cm^−1^ are in good agreement with the existing literature. These peaks at 998 and 568 cm^–1^ may be assigned to the S–S and Mo–S bonds of MoS_2_, respectively [5,22,23,31].

In the spectrum of PPy/MoS_2_ (Figure 1c), the peaks at 3405, 2916 and 2848 cm^−1^ are due to the –OH group of moisture and stretching vibrations of C-H bonds, respectively. In the case of PPy/MoS_2_, all the characteristic peaks of PPy can be seen, along with a new peak at 553 cm^−1^ which confirms the presence of MoS_2_ in the nanocomposite. The peaks observed at 1546, 1460, 1306, 1186, 1042, 922 and 788 cm^−1^ in the spectrum of pristine PPy are observed at slightly higher wavenumbers in the case of PPy/MoS_2_ at 1563, 1466, 1319, 1210, 1047, 932 and 791 cm^−1^, respectively. The shifting of characteristic peaks of PPy in the spectrum of PPy/MoS_2_ shows the electronic/synergistic interaction working at molecular levels [31,33]. 

### 3.2. X-ray Diffraction (XRD) Studies

**Figure 2:**
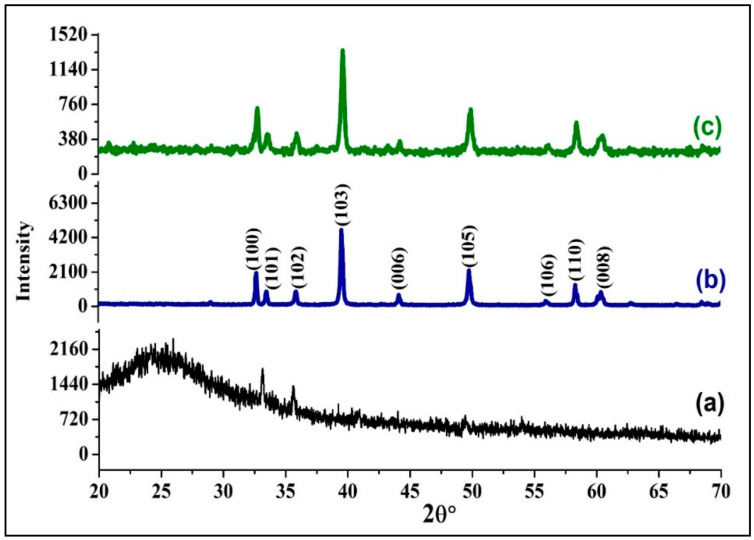
The X-ray diffraction (XRD) spectra of: (**a**) PPy, (**b**) MoS_2_ and (**c**) PPy/MoS_2_.

The XRD spectra of PPy, MoS_2_ and PPy/MoS_2_ nanocomposites are depicted in Figure 2. In the spectrum of PPy (Figure 2a), the broad diffraction peak seen at 2θ = 21–27° shows the amorphous nature of PPy [9,12,31,32]. In the case of MoS_2_ (Figure 2b), the peaks located at 2θ = 32.65°, 33.61°, 35.90°, 39.55°, 44.20°, 49.86°, 56.15°, 58.50° and 60.35° are assigned to (100), (101), (102), (103), (006), (105), (106), (110), (008) and (108) diffraction planes, respectively [20,24,31]. Whereas in the spectrum PPy/MoS_2_ (Figure 2c), the distinctive peaks of MoS_2_ are detected at slightly higher angles, i.e., 2θ = 32.78°, 33.72°, 35.99°, 39.70°, 44.30°, 49.92°, 56.26°, 58.59° and 60.65° respectively, displaying molecular interaction between PPy and MoS_2_. The intensity of all the peaks of MoS_2_ is decreased considerably in PPy/MoS_2_, clarifying the successful and comprehensive coverage of MoS_2_ sheets by the PPy matrix, which could be understood by SEM and TEM images of PPy/MoS_2_.

### 3.3. Morphological Studies

**Figure 3:**
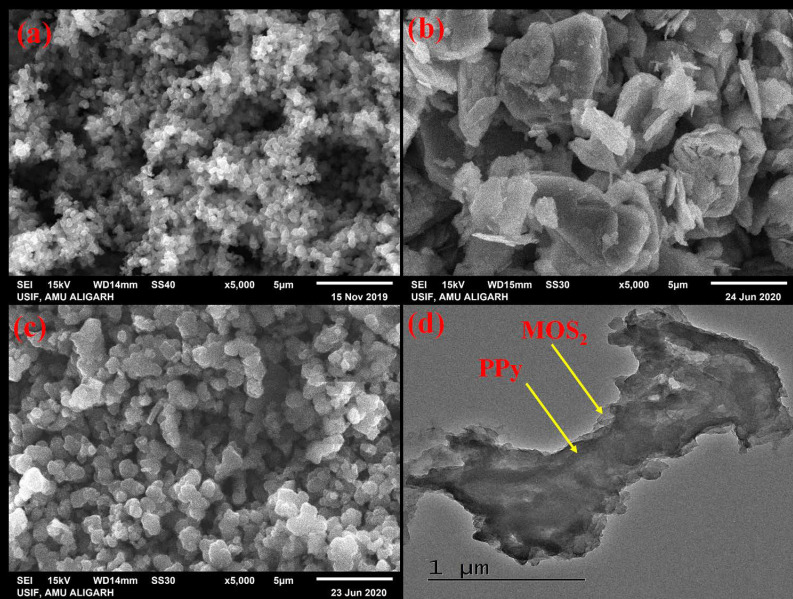
Scanning electron microscopy (SEM) micrographs of: (**a**) PPy, (**b**) MoS_2_ (**c**) PPy/MoS_2_ and (**d**) transmission electron microscopy (TEM) micrograph of PPy/MoS_2_.

The SEM micrographs of pristine PPy, MoS_2_ asnd PPy/MoS_2_ nanocomposites together with the TEM image of PPy/MoS_2_ are presented in Figure 3. The surface of pristine PPy (Figure 3a) consists of globular nanoparticles which are agglomerated with each other [12]. In the case of MoS_2_ (Figure 3b), surface morphology is sheet-like. The SEM image of PPy/MoS_2_ (Figure 3c) shows that pyrrole is successfully polymerized on the surface of MoS_2_ nano-sheets. The size of globular nanoparticles of PPy/MoS_2_ are greater than the size of pristine PPy. Some sheet-like structures are also observed in PPy/MoS_2_, which confirms the presence MoS_2_ in nanocomposites on which pyrrole polymerized. The TEM image of PPy/MoS_2_ is shown in Figure 3d. 

### 3.4. DC Electrical Conductivity

**Figure 4:**
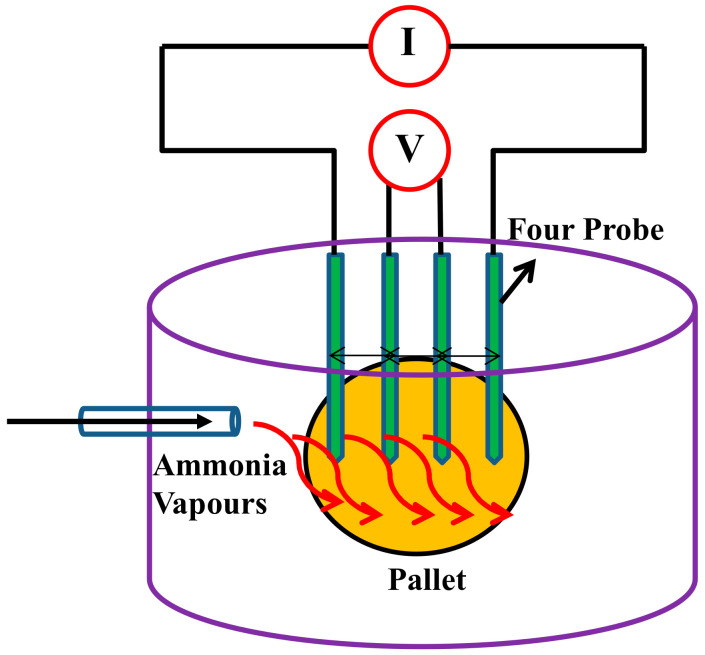
Ammonia sensor unit by the four in-line probes method.

DC electrical conductivity as well as ammonia sensing experiments were carried with four in-line probe instruments attached with a PID-controlled oven manufactured by Scientific Equipment, Roorkee, India. The schematic representation of instrument is shown below in Figure 4.

**Figure 5:**
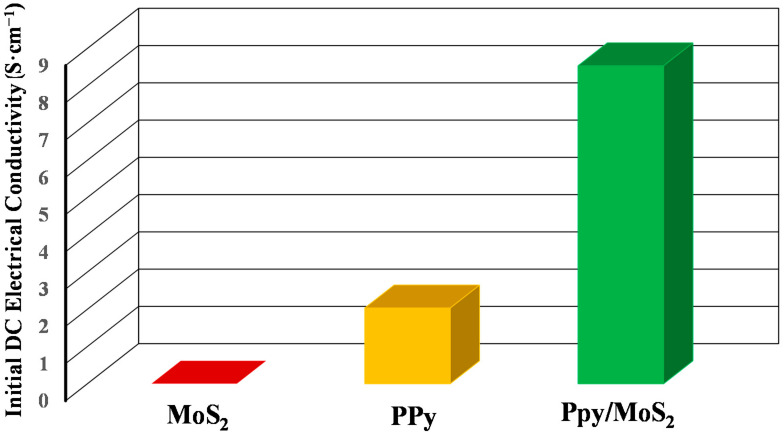
Initial DC electrical conductivities of MoS_2_, PPy and PPy/MoS_2_ nanocomposites.

The initial DC electrical conductivities of MoS_2_, PPy and PPy/MoS_2_ nanocomposites were determined by a standard four in-line probes method, as shown in Figure 5. The electrical conductivities of MoS_2_, PPy and PPy/MoS_2_ nanocomposites at room temperature were observed to be 2.2 × 10^−6^, 2.03 and 8.33 S·cm^−1^, respectively. The electrical conductivity of PPy/MoS_2_ nanocomposites increases drastically after incorporation of MoS_2_ into pure PPy. This sudden rise in electrical conductivity of PPy/MoS_2_ nanocomposites may be due to the interaction of lone pairs of nitrogen of polypyyrole with molybdenum of MoS_2_ producing more holes in PPy, leading to a rise in electrical conductivity. 

### 3.5. Sensitivity

**Figure 6:**
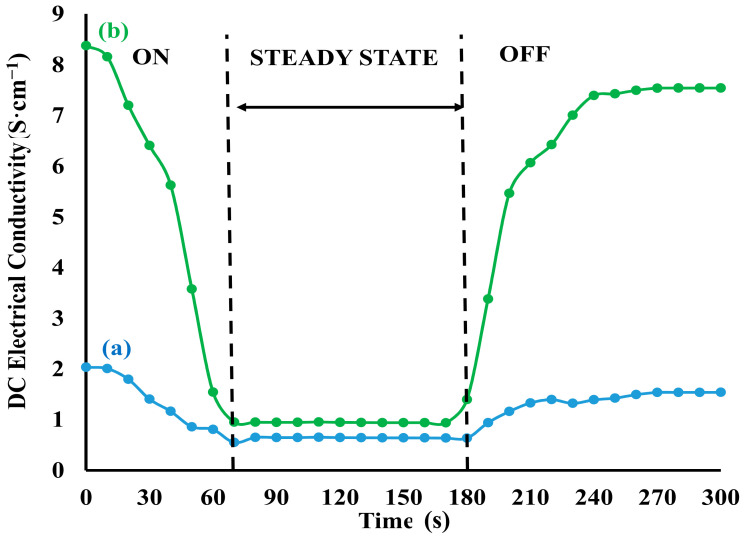
Effect on the DC electrical conductivity of (**a**) PPy and (**b**) PPy/MoS_2_ nanocomposites upon exposure to (1000 ppm) ammonia vapors followed by exposure to ambient air with respect to time.

The sensitivities of PPy and PPy/MoS_2_ towards ammonia vapors with time were studied as shown in Figure 6. Upon passing 1000 ppm of ammonia vapors to the surface of PPy/MoS_2_, the DC electrical conductivity of PPY/MoS_2_ decreased from 8.3719 to 0.9488 S·cm^−1^ in 70 s and reached a steady-state level in ammonia atmosphere for 180 s. After 180 s, as the PPY/MoS_2_ placed in ambient air, the DC electrical conductivity started to increase rapidly and resumed back to 7.5369 S·cm^−1^ in the next 120 s. Similarly, in case of pristine PPy, the change in DC electrical conductivity was found to be very marginal upon exposure to 1000 ppm of ammonia. The excellent sensitivity of PPy/MoS_2_ may be attributed to high surface area and active sites available for more adsorption and desorption for analyte gas.

### 3.6. Limit of Detection

**Figure 7:**
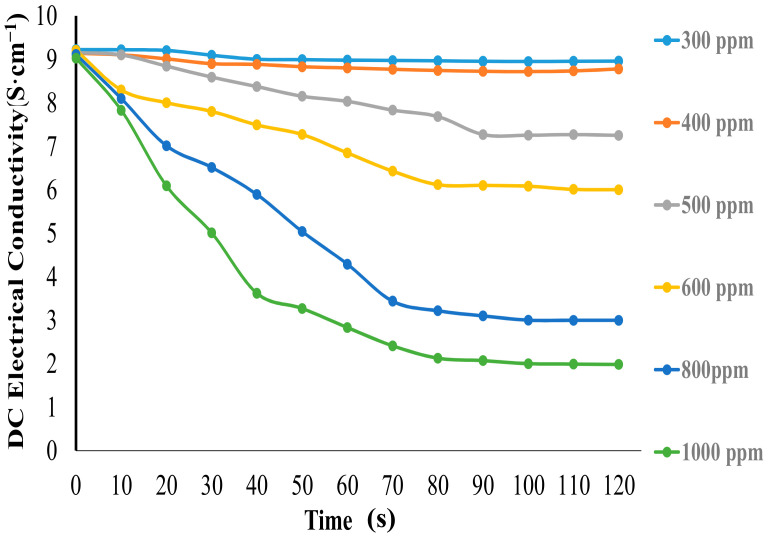
Effect on the DC electrical conductivity of the PPy/MoS_2_ nanocomposites upon exposure to ammonia vapors at different concentrations.

The PPy/MoS_2_ nanocomposites were tested for limit of detection towards various concentrations of ammonia, as shown in Figure 7. As the concentration of ammonia decreased from 1000 to 300 ppm, the rate of change of conductivity also decreased and became minimized for the 300 ppm concentration of ammonia. Thus, the electrical conductivity change of PPy/MoS_2_ nanocomposites could be observed in contact with ammonia as low as 300 ppm concentration.

### 3.7. Reversibility

**Figure 8:**
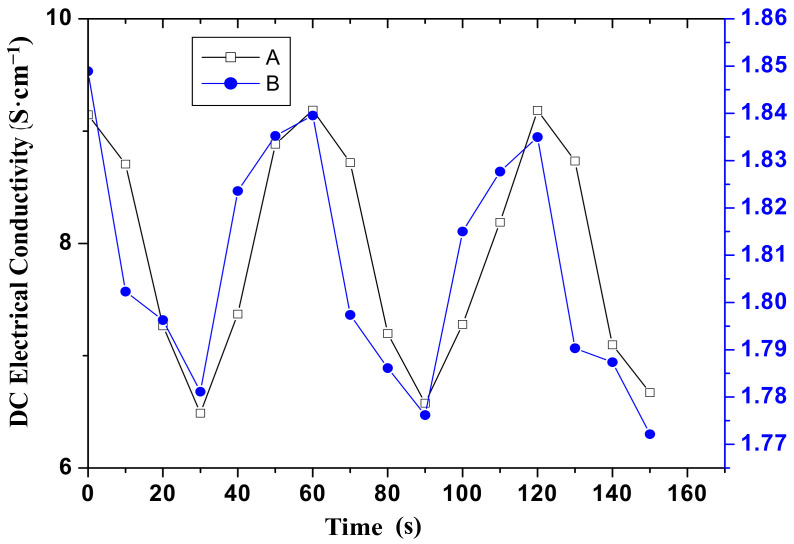
Electrical conductivity of curve A PPy/MoS_2_ nanocomposite and curve B PPy upon alternate exposure to 1000 ppm of ammonia vapors and air with respect to time.

The reversibility of PPy/MoS_2_ nanocomposites and PPy was studied in terms of the DC electrical conductivity as the cycling between the analyte and ambient air without any loss in its sensing performance, as shown in Figure 8. The reversibility of PPy/MoS_2_ nanocomposites and PPy was examined by first exposing the sample to 1000 ppm of ammonia vapors for 30 s, followed by 30 s in ambient air for a total duration of 150 s. Figure 8A shows the reversibility of PPy/MoS_2_ nanocomposites with a fine repeated range of conductivity change from 9.145 to 6.488 S·cm^−1^ in the ambient air and upon exposure to 1000 ppm of ammonia vapors, respectively. The overall conductivity change for PPy/MoS_2_ nanocomposites was about 2.65 S·cm^−1^. Figure 8B shows the reversibility of pure PPy, within the conductivity range of 1.79 to 1.848 S·cm^−1^. The overall conductivity change for PPy was about 0.058 S·cm^−1^, which is very nominal in comparison with PPy/MoS_2_ nanocomposite. There were about 55 times better variations observed in conductivity of PPy/MoS_2_ nanocomposite than that of pure PPy.

### 3.8. Selectivity

**Figure 9:**
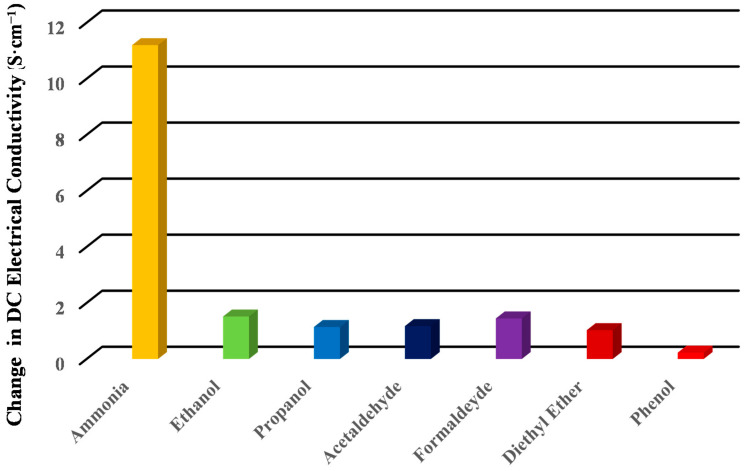
Selectivity of PPy/MoS_2_ nanocomposite toward 1000 ppm of ammonia against different 1 M VOCs.

The DC electrical conductivity responses of PPy/MoS_2_ nanocomposite towards ammonia and VOCs (volatile organic compounds) viz. ethanol, propanol, acetaldehyde, formaldehyde, diethyl ether and phenolat room temperature (~27 °C), are shown in Figure 9. It was observed that the PPy/MoS_2_ nanocomposite showed the highest change in DC electrical conductivity towards 1000 ppm of ammonia among different analytes. Such a high selectivity of ammonia results from the highest electron donating tendency among the analytes tested. Therefore, the lesser electron-donating affinity of VOCs causes a lesser conductivity change. This also infers the higher selectivity of the PPy/MoS_2_ nanocomposite toward NH_3_.

### 3.9. Sensing Mechanism

**Figure 10:**
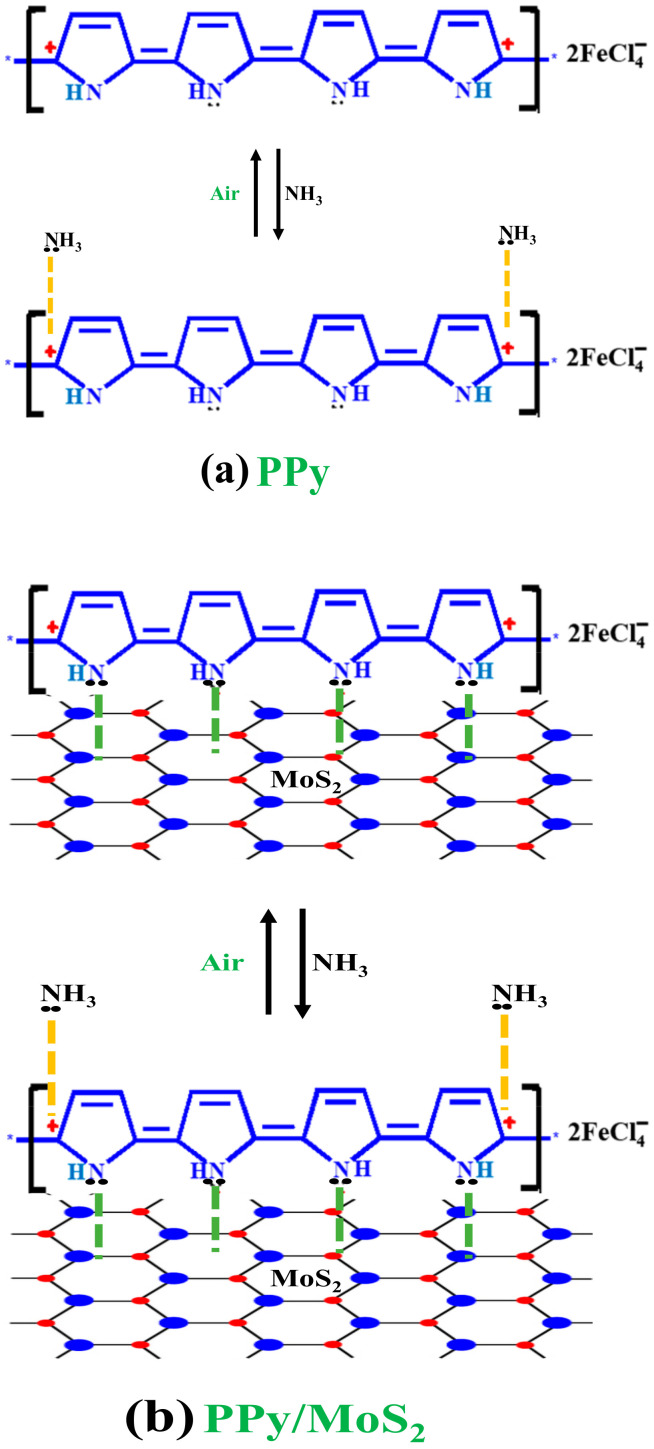
Schematic representation of the plausible NH_3_ adsorption-desorption sensing mechanism on (**a**) polypyrrole (PPy) and (**b**) Polypyrrole/MoS_2_ (PPy/MoS_2_) nanocomposites.

The ammonia sensing mechanism of PPy and PPy/MoS_2_ nanocomposites is simply explained on the basis of change in electrical conductivity upon adsorption and desorption of analyte at room temperature, as shown in Figure 10a,b. PPy, being a p-type doped conducting polymer, interacts with lone pairs of ammonia which resist the mobility of polarons of PPy, resulting in a decrease in electrical conductivity [9,12]. In case of PPy/MoS_2_, the emergence of high electrical conductivity may be due to the interaction of lone pairs of nitrogen of polypyyrole with molybdenum of MoS_2_, producing more holes in PPy, leading to a rise in electrical conductivity. As the number of holes increase on PPy, it creates more interaction sites on the polymer with ammonia, leading to high sensitivity of the PPy/MoS_2_ nanocomposite. 

The positive charges on the polypyrrole backbone are polarons (Figure 10a). The main charge carriers in such materials are polarons, besides, protons may also participate in electrical conduction to a very small extent. The main reason for a decrease and increase in electrical conductivity is due to chemisorptions and desorption of ammonia respectively, involving the interaction between the lone pair of ammonia and positive polarons on the polypyrrole backbone. The results show that there is no chemical reaction between ammonia and polypyrrole leading to complete reversibility of electrical conductivity due to un-doping of the polymer [12,30].

As soon as the PPy/MoS_2_ nanocomposite is exposed to ambient air, the ammonia molecules get completely detached from the polarons of the PPy/MoS_2_ nanocomposite and therefore, the electrical conductivity reverts almost to its initial value (Figure 10b). 

The sensing performance of PPy/MoS_2_ nanocomposite was compared with other reported work, as shown in Table 1.

## 4. Conclusions

PPy and PPy/MoS_2_ nanocomposites were successfully synthesized via a chemical oxidative polymerization route. FTIR, XRD and morphological studies proved that the MoS_2_ is completely incorporated into the PPy matrix. The PPy/MoS_2_ nanocomposite showed very high DC electrical conductivity compared to that of pure PPy. The gas sensing results revealed that the PPy/MoS_2_ nanocomposite was found to be an outstanding material for ammonia sensing with quick response and decent selectivity against VOCs. There were about 55 times better variations observed in conductivity of PPy/MoS_2_ nanocomposite than that of pure PPy during sensing. Therefore, the PPy/MoS_2_ nanocomposite may be a worthwhile, proficient and promising material for ammonia sensing.

## Figures and Tables

**Table 1:** Sensing performance of this work (PPy/MoS_2_) compared with reported work.

Ammonia Sensing Properties	Co_3_O_4_/SnO_2_ [35]	MoS_2_/ZnO [36]	MOx (SnO_2_, CuO) Modified rGO [37]	TeO_2_ [38]	SnO_2_/SWNTs [39]	PANI/α-Fe_2_O_3_ [40]	PPy/MoS_2_ (This Work)
Response Time	4 s	10 s	100–120 s and 108–132 s	3.1 min	100 s	75 s	60–70 s
Reversibility	17 s	11 s	98–109 s and 98–138 s	5.6 min	3.2 min	5 s	50–60 s
Operating Temperature	200 °C	Room Temperature(RT)	RT	170 °C	RT	RT	RT
Selectivity	Very good	Excellent	Not reported	Not reported	Not reported	Good	Very good
